# Editorial: Exploring the role of exosomes in disease progression and therapeutics in neurodegeneration

**DOI:** 10.3389/fnagi.2023.1177063

**Published:** 2023-03-28

**Authors:** Arif Tasleem Jan, Safikur Rahman, Diana K. Sarko, Elrashdy M. Redwan

**Affiliations:** ^1^Gene Expression Lab, School of Biosciences and Biotechnology, Baba Ghulam Shah Badshah University, Rajouri, India; ^2^Department of Botany, Munshi Singh College, Babasaheb Bhimrao Ambedkar Bihar University, Muzaffarpur, Bihar, India; ^3^Department of Anatomy, Southern Illinois University School of Medicine, Carbondale, IL, United States; ^4^Biological Science Department, Faculty of Science, King Abdulaziz University, Jeddah, Saudi Arabia; ^5^Protein Research Department, Genetic Engineering and Biotechnology Research Institute, City of Scientific Research and Technological Applications (SRTA-City), New Borg EL-Arab, Alexandria, Egypt

**Keywords:** exosomes, neurodegenerative diseases, protein aggregation, protein folding, proteinopathies

## Introduction

Neurodegenerative diseases (NDs; including Alzheimer Disease, Parkinson Disease, Amyotrophic lateral sclerosis, Huntington Disease, and others) are characterized by a progressive loss of neuronal cells and/or function that often leads to physical disabilities and in some cases progresses to death. Being localized to specific parts of the brain or showing systemic distribution, the progression of NDs is irreversible and often incurable as the progression mechanisms show variation and are not fully understood. Of the different risk factors associated with NDs, age along with prolonged processes of inflammation and oxidative stress are considered the major contributing factors. Considering the elusive nature of NDs, studies are currently directed at exploring and understanding disease development mechanisms, factors affecting disease progression, accurate diagnosis, timely interventions, and the development of suitable therapeutics that could combat the occurrence of the disease.

The recent trend in mechanistic foci for proper diagnosis and optimal treatment strategies includes studies oriented toward exploring the role of exosomes in disease progression and therapeutics of NDs. Extracellular vesicles (EVs) including exosomes are membrane-enclosed entities that display specific components on their surface and sequester molecules such as nucleic acids to shuttle as cargo intracellularly for communication purposes. It has been found that their production and transport *via* body fluids such as blood, breast milk, etc., influence the physiological and pathological parameters within the system. Studies performed with respect to their composition, biogenesis, and trafficking have provided deeper insights into the marked changes they undergo in the context of different human diseases.

The main goal of this Research Topic was an exploration of the latest outstanding discoveries pertinent to different aspects of NDs and insights into emerging diagnosis and treatment strategies. The present Research Topic includes six review articles that provide in-depth insight into different aspects of EVs in particular exosomes with respect to their composition, biogenesis and trafficking, their diagnostic utility, and target-specific delivery of therapeutic modules. The articles published in the issue are directed toward exploring the role of EVS in the improving cognitive function, in addition to covering various aspects of disease progression and therapeutic delivery in different NDs.

The review “*Extracellular Vesicles: A New Paradigm in Understanding, Diagnosis and Treating Neurodegenerative Diseases*” by Dar et al. illustrated how NDs are becoming one of the leading causes of disability and deaths internationally. The article highlighted an understanding of the role of exosomes in the pathogenesis and progression of NDs. With respect to the challenges posed by delivering drugs across the biological barriers, this review provided information about the utility of EVs in delivering therapeutic modules particularly non-coding RNAs across the blood-brain barrier to the central nervous system.

The review by Weng et al., “*Role of Exosomes as Mediators of Neuroinflammation in the Pathogenesis and Treatment of Alzheimer's Disease*,” summarized the role of neuroinflammation in the pathogenesis of Alzheimer's disease. Microglial cells play a significant role in the pathogenesis and secretion of tau in EVs in particular exosomes. As an inflammatory mediator, EVs carrying tau, Aβ, and other entities, induce neuroinflammation through diffusion at the interconnected neurons. The authors reported an association of neuroinflammation and EVs particularly exosomes with abnormal protein aggregates and neuronal death that proceeds with the development and severity in the pathogenesis of Alzheimer's disease.

The review “*Evolving role of Extracellular Vesicles (Exosomes) as Biomarkers in Traumatic Brain Injury: Clinical Perspectives and Therapeutic Implications*” by Khan et al. explains the methodology for identifying biomarkers for different NDs. In their study, the authors emphasized the role of the exosome-mediated dissemination of protein cargo implicated in the progression of traumatic brain injury. Compared to the short half-life of proteomic blood-based biomarkers, exosome-based protein biomarkers offer the advantage of generating a better understanding of cellular damage and neuroinflammation in the context of traumatic brain injury and other NDs.

The review by Duggan et al., “*Exosomes in Age-related Cognitive Decline: Mechanistic Insights and Improving Outcomes*,” discussed the potential of exosomes in age-related cognitive decline. The authors summarized information on recent developments in different approaches (genomics, proteomics, and functional imaging) toward delineating cognitive decline in age-related neuropathologies. The study focused on the critical role of the cargo, particularly miRNA and aggregate-prone proteins, in regulating intercellular communication. This offered a novel tool for predicting cognitive decline among the elderly in an accurate and reliable manner in addition to highlighting the importance of nanocarriers in delivering therapeutics to the brain.

The review “*Mesenchymal Stem Cells and Exosomes Improve Cognitive Function in the Aging Brain by Promoting Neurogenesis*” by Zhang et al. discussed brain aging as a critical biological process that affects the physiological balance between health and disease states. Aging in the form of degenerative changes in structures and decline of function manifest as diminished adaptability and resistance within the human body. The authors elaborated on the role of mesenchymal stem cells–and exosomes derived from them in combatting aging by promoting neurogenesis *via* improvement in brain function and subsequent decline in neuroinflammation.

Wang et al., in their study titled, “*Adipose-Derived Mesenchymal Stem Cells Combined with Extracellular Vesicles May Improve Amyotrophic Lateral Sclerosis*” discussed the role of adipose-derived mesenchymal stem cells and EVs preferably exosomes in neuronal injuries. In their study, the authors presented details on the use of adipose-derived mesenchymal stem cells and EVs as potential interventional agents in the treatment of injured nerves among individuals suffering from Amyotrophic Lateral Sclerosis. Additionally, this review highlights the therapeutic potential of adipose-derived mesenchymal stem cells and the protective effects of EVs concerning the pathogenesis of Amyotrophic Lateral Sclerosis and expounds on their practical utility in clinical settings.

## Conclusion

The articles published in this Research Topic collectively summarize the role of EVs in diagnostic and therapeutic pursuits. Upholding a great promise in paving the way for strategic development of diagnostic methods, cargo's in particular tau, Aβ and others loaded as payload to EVs, is beneficial toward achieving accurate diagnosis of the different NDs. Progress in the EVs research has deepened our understanding about their utility in delivering different therapeutic payloads such as small molecules, non-coding RNAs (siRNA), etc, to improve the brain function. The translational opportunity offered by EVs reflecting their utility in the diagnosis and therapeutics needs improvement and expansion in terms of scalability, efficient drug loading and targeting. A lot of new start-ups in particular Evox therapeutics (United Kingdom), ExoPharm (Australia), and Codiak Biosciences (USA) have recently started their exploration toward extending the possibility of the use of EVS in diagnosis and in achieving target specific delivery of drugs. Despite significant progress, studies need to be focused on their production in different cellular backgrounds, developing effective methods of isolation along with the purification procedures, scalability, efficient drug loading, specific targeting, increasing bioavailability and most importantly cost-effectiveness to achieve practical applicability of their use in clinics for diagnosis and effective treatment for broad range of neurodegenerative diseases.

## Author contributions

ATJ and EMR prepared the initial draft of this editorial. ATJ, SR, DKS, and EMR carefully revised the draft. All authors contributed to the contents of this article and approved the final version for its submission to *Frontiers in Aging Neuroscience* journal.

